# Addictive neurons

**Published:** 2017-01-30

**Authors:** Sodikdjon A. Kodirov

**Affiliations:** 1 I. P. Pavlov Department of Physiology, State Research Institute of Experimental Medicine, Russian Academy of Medical Sciences, Saint Petersburg 197376, Russia; 2 University of Texas at Brownsville, Department of Biological Sciences, Texas 78520, USA; 3 Johannes Gutenberg University, 55099 Mainz, Germany; 4 Almazov Federal Heart, Blood and Endocrinology Centre, Saint Petersburg 197341, Russia; 5 Neuroscience Institute, Morehouse School of Medicine, Atlanta, GA 30310, USA

**Keywords:** ADP, AHP, amygdala, lateral septum, LTD, LTP, medial prefrontal cortex, paired pulse facilitation, rebound action potential, spike

## Abstract

Since the reward center is considered to be the *area tegmentalis ventralis* of the hypothalamus, logically its neurons could mainly be responsible for addiction. However, the literature asserts that almost any neurons of CNS can respond to one or another addictive compound. Obviously not only addictive nicotine, but also alcohol, amphetamine, cannabis, cocaine, heroin and morphine may influence dopaminergic cells alone in VTA. Moreover, paradoxically some of these drugs ameliorate symptoms, counterbalance syndromes, cure diseases and improve health, not only those related to the CNS and in adults, but also almost all other organs and in children, e.g. epilepsy.

*Without any fear of exaggeration, that the proper use of cocaine, before operations involving the conjunctiva, cornea, or iris, lessens the pain of the patient*^…^

Howe, 1884

## Introduction

Any drug has multiple targets and this also concerns those targeted to the central nervous system (CNS). It is common in the literature that either the agent or its target are always emphasized as prevailingly important depending on research interests ^[1]^. However, conclusive effects are not always observed for classical agents, except perhaps for acetylcholine ^[2]^. The complexity of targets and the interrelationship among pathologies ^[3, 4]^ make the brain even more complex. Although there is a reward center – *area tegmentalis ventralis* – in CNS, neurons and their neurotransmitters are influenced by addictive substances in several other regions and also contribute to the underlying addictive behaviors ^[5]^.

The ventral tegmental area (VTA) in turn may not only process and weigh the reward expectancy, but as recognized earlier by electrical stimulation with electrodes, this nucleus may contribute to the ‘expression of unpleasant emotion’ ^[6]^. The center of emotions thereafter was established to be the amygdala, but the importance of this region meanwhile is almost replaced by the frontal or prefrontal cortex – PFC. Moreover, the anxiety is not represented by the amygdala alone, but lateral septum – LS may also be involved ^[7]^. Many syndromes comprise changes in electrical properties of neuronal membranes that is either small (depolarization) or with more impact (spikes). Although neural cells in CNS of Animalia have similar electrical properties in the form of spikes (either spontaneous or triggered), only neurons of humans are able and will respond to ‘clench your hand to make a fist!’ ^[8]^.

Human beings have multiple choices when it concerns addictive substances ranging from the traditionally accepted to those (still) prohibited ones. These are alcohol, amphetamine, cocaine, marijuana, tobacco and in lesser extent other popular drugs. All these agents are also the subject of ongoing research. The targeted research in endogenous cannabinoids (eCB) field started with the discovery of arachidonylethanolamide – AEA ^[9]^. The biological actions of eCBs target the binding to the type 1 and 2 cannabinoid receptors (CB_1_R and CB_2_R). The native ligands of eCBs are derivatives of arachidonic acid. The CB_1_R is mainly expressed in the CNS while CB_2_R in peripheral tissues. The eCBs also participate in reward mechanism/cascade of the brain.

The AEA known as ‘anandamide’ was for the first time identified in the *vas deferens* of mice. The electrically evoked twitch response is blocked by this ligand, similar to those of psychotropic cannabinoids ^[9]^. The anandamide is an endogenous cannabimimetic eicosanoid and this name derives from the Sanskrit word *ananda* meaning ‘bliss’. The marijuana *Cannabis sativa* (Linnaeus 1753) effects are mimicked by anandamide in the brain’s reward circuits. Addicted individuals may want to quit but the addiction trace will not cease easily. Earlier on the severity of withdrawal effects of addictive drugs was recognized and correlations with EEG patterns are documented ^[10]^.

Among all addictive drugs only activity of eCBs and receptors can be distinctly demonstrated by a designated electrophysiological paradigm – depolarization-induced suppression of excitation (DSE) and inhibition (DSI) in many brain regions ^[11]^. Note that these phenomena were known before and could predict the exact machinery, since ‘DSI is due to somatodendritic exocytosis of a retrograde messenger, and that this exocytosis is highly sensitive to [Ca^2+^]_i_’ ^[12]^ in Purkinje neurons.

The aim of this work is not to review all existing studies (which is not possible anymore), but rather to exemplify the duality of the effects of substances and that there are no distinct neurons that are solely prone to addiction.

### VTA DA neurons

Neurons of VTA are of two major subtypes – DA (dopamine) and GABAergic. These two types of cells as constituents of the ‘ventral tegmental area microcircuit model challenges the classical view that GABA neurons exclusively reduce dopamine neuron firing and bursting’ ^[13]^. DA and GABAergic cells are distinguished by both action and membrane potential (AP and MP) properties as the former exhibits robust sag that is absent in the latter ^[14]^. VTA DA neurons express HCN and Kv – voltage dependent K^+^ channels similar to other excitable cells ^[15]^ and the *I*_h_ (see Figure 5) and their tails are inhibited by 20 μM DA ^[16]^. The amplitude and density of *I*_A_ is higher in DA *vs*. GABAergic neurons, but the conductance of channels are comparable ^[14]^. The respective values of the recovery from inactivation also differ and are ~50 *vs*. 300 ms in young rats. There are also SK channels in VTA DA neurons and their outward K^+^ currents are decreased by 15 μM NMDA ^[17]^ revealing the complex nature of neurons and all elements of their membrane. This is also substantiated by their spiking with common basic properties yet distinct rates and modes.

**Figure 1:**
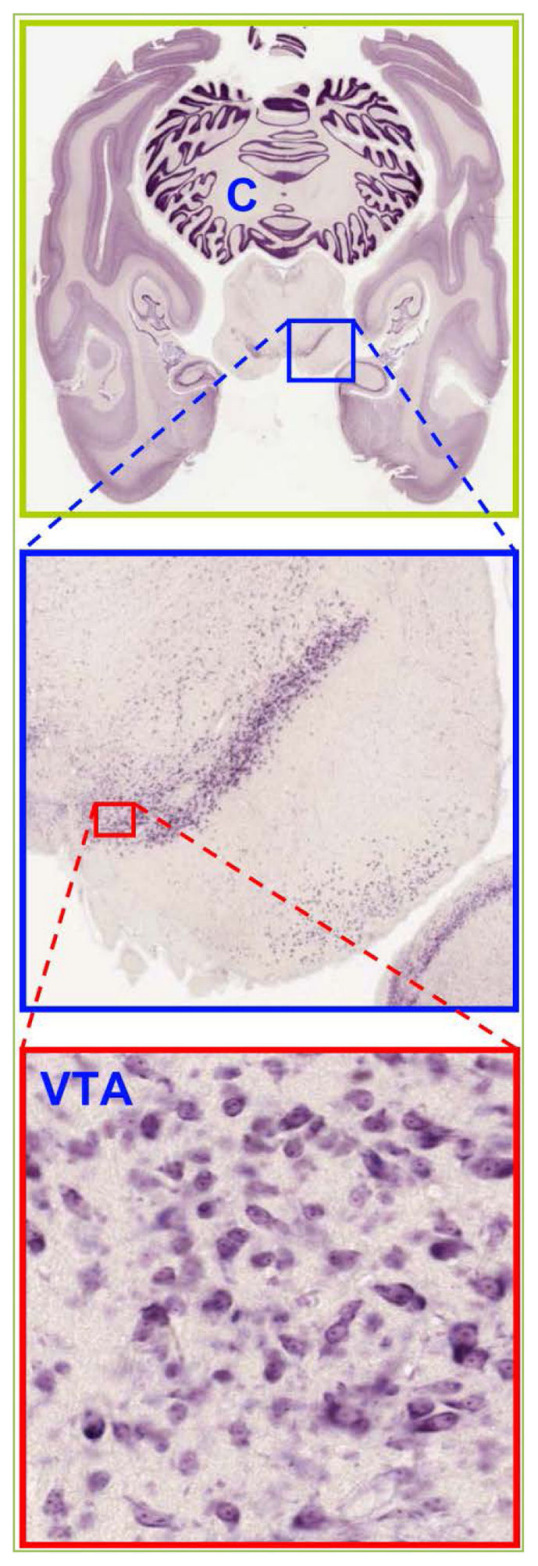
VTA of rhesus monkey *Macaca mulatta* Both hemispheres of the brain are shown in horizontal plane (www.brainmaps.org). Note that this sectioning enables inclusion of more nuclei in a single slice than the coronal one (see Figure 2). Specifically, also the C – cerebellum is present. Reprinted with permission ^[123]^.

**Figure 2:**
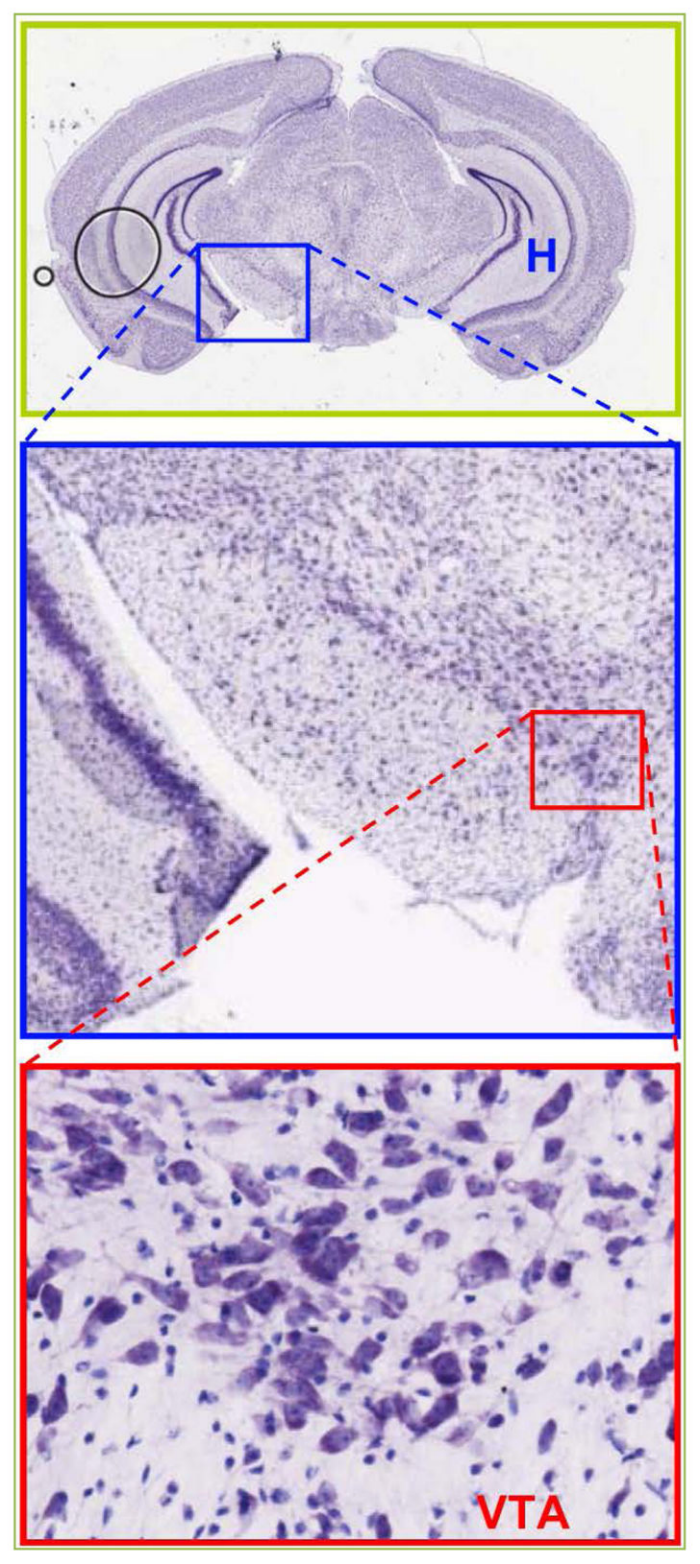
VTA of mouse *Mus Musculus* Coronal section of the brain containing several regions including H – hippocampus. VTA is shown at higher magnification (www.brainmaps.org). Reprinted with permission ^[123]^.

There is no a strict area division between the VTA and SNc – *substantia nigra pars compacta* nuclei (Figure 1 and 2) and DA neurons resemble to a greater extent each other with common electrophysiological hallmarks. DA neurons of SNc have a depolarized RMP – resting membrane potential and in order to repolarize to −60 mV a negative current of ~65 pA is injected ^[18]^. Upon the hyperpolarizing steps of −90 pA these neurons exhibit a sag (see Figure 4) with a maximal amplitude of ~20 mV during 1 s. No gap junction response was observed among the pairs of DA neurons, but there was either a train of APs or a single spike-evoked hyperpolarization (SEH) that was heterogeneous in terms of amplitudes. As expected the neurotransmitter between DA neurons is dopamine, however perhaps not alone, since neither 300 μM Cd^2+^ (targets Cav – voltage dependent Ca^2+^ channels) nor the antagonist of type 2 dopamine receptor (D_2_R) raclopride at 1 μM could obliterate the SEH. When MP is held at −75 mV (by −95 pA) 50 μM ZD7288 does not alter evoked APs waveforms and rates in neuron 1 and related SEH in postsynaptic cells held at −80 mV (−220 pA). Interestingly, ‘once cell2 had been depolarized at −60 mV, ZD7288 became effective and abolished the SEH’ ^[18]^. This is indeed an important result, but the MP of 2^nd^ cell is denoted to be −85 mV and also considerably less current was injected (−90 *vs*. −220 pA). Similarly it is confusing why the MP of active neuron was changed to −90 mV that now was achieved by only −20 pA when under control conditions −95 pA was necessary to hold MP at −75 mV. Therefore, perhaps not effects of ZD7288, but the appearance of SEH is voltage dependent as also revealed by authors. Since these are similar DA neurons holding the MP at identical −60 mV would be comprehensive as it is shown during recording of sag and its abolishment in the same 2^nd^ cell. Consistently, the rebound action potential – RAP – is obliterated by ZD7288 ^[18, 19]^. Note that as highlighted the ZD7288 exerts heterogeneous effects on neurons ^[19]^.

**Figure 3:**
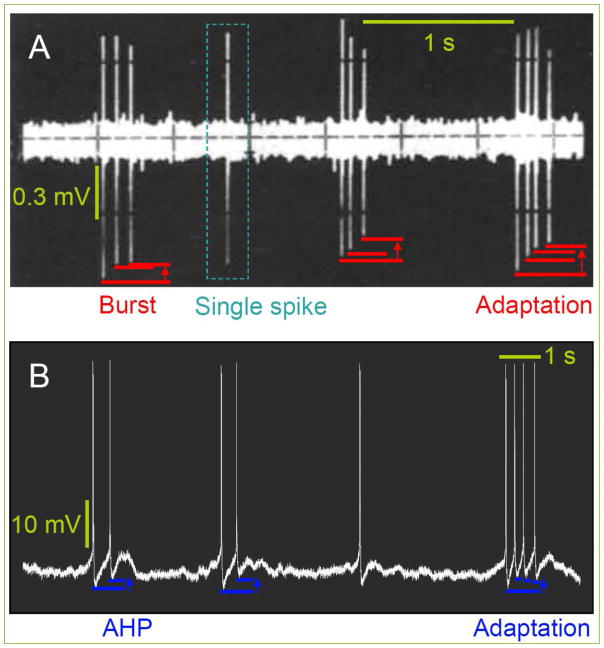
Spontaneous bursting (A) Single spike and bursts in rat VTA DA *in vivo* ^[24]^. There is an adaptation of amplitude during the bursts, but note that the same is not always observed at opposite phases of spikes. (B) Spiking in rat LS neuron in whole-cell mode after nicotine *in vitro*. Amplitudes at overshoot are similar but there is a decrease at negative potential and it concerns the AHP as elucidated earlier. Reprinted with permission ^[20]^.

Transition into the bursting mode (Figure 3B) perhaps could be considered as a readout of addictive neurons ^[20]^. VTA DA neurons respond to a synthetic cannabinoid receptor agonist WIN55,212-2 at 1 mg/kg with an increased spontaneous bursting that is quantitatively similar to the effects of 0.125 mg/kg SM-11 ^[21]^. When the concentration of SM-11 were 2 or 4 fold higher, then the bursting rate was considerably decreased compared to basal values hinting at tonic release of eCB. These effects of both compounds were similarly reflected on numbers of spikes within a burst (SWB). Caution: it is not a comparison between WIN and SM-11 but SM-11 was concurrently applied with WIN in order to antagonize the effects of the CB_1_ agonist. Therefore, SM-11 was proposed as a new CB_1_ antagonist. Stimulation of nucleus accumbens – NAc evoked an antidromic spike in VTA DA neuron that had an identical phenotype and similar amplitude to spontaneous ones. The stimulus was ineffective when a neuron experienced a recent spontaneous spike and hence there was a refractory period which presumably is more than 10 ms and perhaps ~40 ms based on the latency of the evoked spike. WIN increased the basal spontaneous frequency from ~3 to 5 Hz. The spike frequency in VTA DA neurons of male rats is similar to those of mice (~3 Hz) of the same gender at P60 ^[22]^. Voluntary drinking in these adolescent males did not alter spike rates. Units of summarized values are not clear, since baseline rates are shown obviously in *Hz* and effects of ethanol at 10, 20 50 mM refers to percentage changes (data most probably is presented as a ratio). Independent what an actual unit of the value of −0.4 is, the frequency was decreased at 10 mM ethanol in naïve males, while increased (9.5) in drinkers. What is the difference between the WT and TH-GFP^+^ mice? The rate in the former ranged between 1 and 12 Hz, while in the latter from 1 to 13 Hz. The rate of spikes decreased at P120 to 2.5 Hz in naïve males. The polarity of effects at 10 mM ethanol was identical to that of P60, but those of 20 and 50 mM were not significant. Comparisons as to age are not entirely adequate as data of P60 comprises the maximum of *n* = 42, while those of P120 *n* = 11, as well as to concentration dependencies, since it was not perhaps always a cumulative application of all three. Also the criteria of 1–12 Hz did not apply to adults.

In adults rats the rate of spikes in VTA DA neurons is ~3 Hz *in vivo* and LSD – lysergic acid diethylamide decreases the number of bursts and SWB starting only at 30 μg/kg while it obliterates at 120 ^[23]^. Spontaneous spikes are not always observed in VTA DA neurons and bursting is even seldom ^[24]^. The particular feature of SWB concerns the gradual decrease of amplitude of each subsequent spike (Figure 3A).

Since VTA in rodents and monkeys has no clear borders (Figure 1 and 2) as a nucleus unlike the amygdala, hippocampus and septum, defining DA neurons would be always ‘somewhat misleading’ by any criteria ^[22]^. Therefore, also previous studies in rats observed heterogeneities in spike properties and when possible have classified them as type 1 or 2 ^[24]^. The latter has considered those triggering antidromic spikes (in response to electrical stimulation) with a latency of ~13 ms as VTA DA neuron – type 1.

As revealed by fast-scan cyclic voltammetry (FSCV) the dopamine concentration in NAc is ~ 17 nM ^[25]^. When the state of inexcitability of DA VTA neurons was achieved by 10 s (not 20 s as stated) stimulation accompanied by a ~1 nA inward current, it declined to ~10 nM and upon the cessation of stimuli it gradually returned back to the baseline. Although, also WT VTA were infected with an adeno-associated viral construct encoding Arch-eGFP, only in TH-Cre mice the above-mentioned effects were observed, since AAV was Cre inducible. Tyrosine hydroxylase – TH targets only neurons releasing catecholamines ‘either into the circulation or locally to brain regions as a response to stress’ ^[26]^ and which ‘comprise epinephrine (adrenaline – A), norepinephrine (noradrenalin – NA), and dopamine (DA)’ ^[27]^.

In WT mice the content of DA has slightly increased to ~ 20 nM ^[25]^ that perhaps comprises basal variations. However, the electrophysiology of APs is not very convincing, since the stimulation paradigm is considerably short (~0.5 *vs*. 10 s). Moreover, since the inter spike interval was on average ~500 ms, what if the stimulation occurs within it, i.e. between the two spontaneous APs? Strictly speaking then ‘spikes / second’ is not correct. Note that the DA content in response to evoked release by electrical stimulus is higher and reaches ~55 nM in TH-Cre. During the Cre-inducible inhibition the peak concentration was ~30 nM. The latter hints that the silencing might be effective only toward the baseline neuronal activities. This procedure increased the number of *fos* expressing neurons in basolateral amygdala (BLA) from ~55 to 105 per 1 mm^2^ in TH-Cre mice. Effects were not restricted to only stimulated sides in BLA and the septum in contrast to the hypothalamus. Significant *fos* expression is observed in the lateral nuclei of septum (LS) compared to WT mice. No apparent changes are seen in the medial septum (MS) of bothgroups of mice.

Dopamine has an effect whether direct (via dedicated receptors) or indirect and is not unique to mammals, but universal to all animals ^[28]^. DA receptor expression in HEK293 cells is accompanied by an increase in cAMP. The *EC*_50_ of DA was 35 nM while that of apomorphine 552 nM. The 37% of amino acid sequence of type 1 dopamine receptors (D_1_R)were identical in human and mollusks.

### Alcohol

Alcoholism is more common than dependence to any of drugs of abuse and easily revealed in a family and friends settings or public places. Basic research in this field involves the exposure of rodents to ethanol. The concentration of ethanol used in some studies reaches often a 100 mM range, which seems too high compared to other data obtained from the ‘middle one-third to two-thirds of the hippocampal’ molecular layer – ML ^[29]^. The tonic GABAergic currents in WT ML interneuron were increased by ~6 pA after application of 30 mM ethanol that was ineffective in Gabrd^−/−^ mice –δ subunit null mutant. The properties of cells were not apart between WT and KO male animals including the cumulative probabilities of inter-event intervals. However, this plot and these curves have shortcomings. The scale does not reflect the usual logarithmic distribution of values and the interval should read *s* instead of *ms*. Curves are very different from commonly observed appearances ^[11]^ and by comparing only two cells it is hard to establish similarities between two groups. This notion could be supported by differences in frequencies of IPSCs – inhibitory postsynaptic currents in ML interneurons of Gabrd^−/−^ and WT mice under control conditions with values of ~18 *vs*. 12 Hz for an equal *n* = 6 and even it was not significant because of SEM of ~6 *vs*. 0.6 Hz. Nevertheless, it was concluded that the ‘ML interneurons express a novel and functional GABA_A_R partnership composed of α1βxδ subunits that mediates their tonic inhibitory currents and is modulated by sobriety-impairing concentrations of ethanol’ ^[29]^.

**Figure 4:**
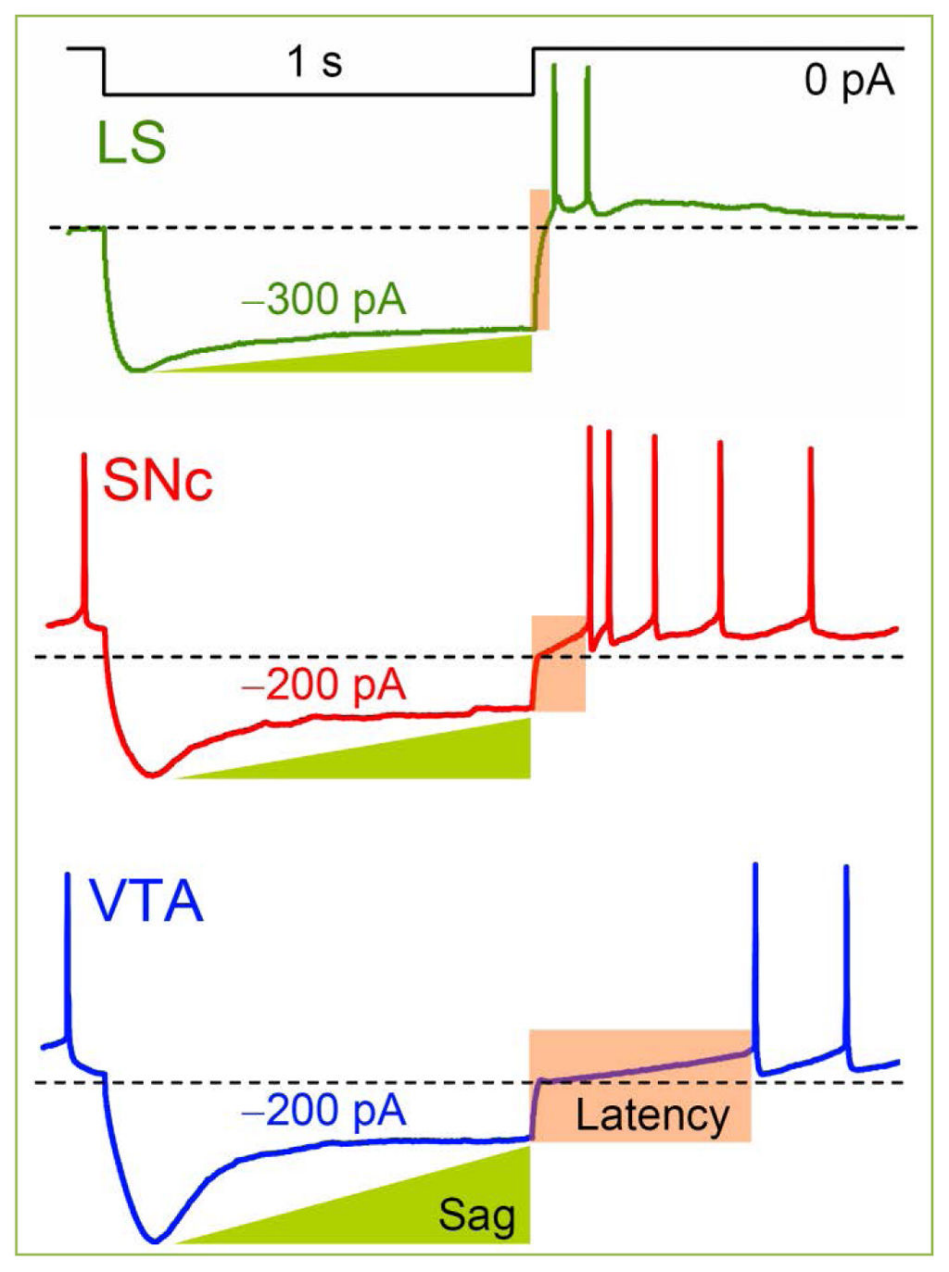
Sag and RAP Properties of MP and APs are different in LS, SNc and VTA neurons. LS neuron is silent at RMP ^[19]^ while cells in SNc and VTA are spontaneously active as can be appreciated by APs prior to hyperpolarizing steps. Note the progressive increase in sag (despite lesser current magnitude of −200 *vs*. 300 pA) and latency to 1^st^ RAP (see also the text). The AHP in SNc is of highest amplitude at 1^st^ RAP and gradually decreases thereafter while in LS and VTA it is relatively constant. Dashed lines closely reflect the RMP with values of −59 mV in LS and an equal −50 mV in SNc and VTA. Reprinted with permission ^[30]^.

**Figure 5:**
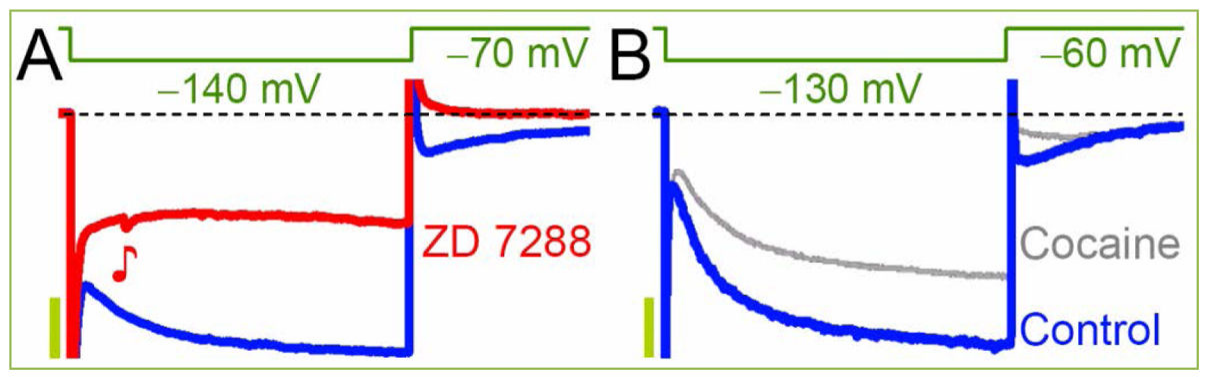
Comparison of *I*_h_ in the brain (A) Inward currents in rat LS neuron before and after 10 μM ZD 7288 ^[19]^. Note the obliteration of tail current. (B) Currents waveform in VTA DA neurons from rats treated with saline (Control) and sensitized to cocaine. The *I*_h_ in VTA are more pronounced and activate faster than in LS. Cocaine influences only the peak tail currents. This is expected, since ZD completely blocked the *I*_h_ in LS, while cocaine only decreased it significantly in VTA. Furthermore, currents in the presence of cocaine still resemble the *I*_h_ but after ZD there is no a sag counterpart. Occasional PSC – postsynaptic current (♪) validates both the cell and slice qualities. Pulse durations are identical at 1 s and the respective amplitude scales are 120 and 200 pA. Reprinted with permission ^[92]^.

In P15 rats VTA DA neuron 55 mM ethanol accelerates the rate of spontaneous APs that does not involve the depolarization of MP as it can be judged by unchanged thresholds before and during application and wash-out ^[30]^. Effects were mainly targeted to a decrease in after-hyperpolarizing potential (AHP), but also accompanying changes in the magnitude of overshoot potential are observed. All these changes were substantially reversible. This concentration also increased the *I*_h_ measured upon the hyperpolarization to −125 mV from HP of −45 mV without influencing the waveforms. Normalized conductance revealed a similar right shift by ~4 mV in DA neurons of both SNc and VTA, perhaps therefore data are presented combined. Interestingly, conductance values in control and ethanol groups are not apart in the range of −125 and −85 mV at which the activation of HCN channels are maximal. DA neurons in these two nuclei can be distinguished only by delays in RAPs (~300 *vs*. 800 ms, Figure 4), since all other active and passive parameters are comparable. RAPs are sensitive to the magnitude of sag and not every neuron exhibits them, sometimes there is only a rebound tail potential – RTP ^[19]^. Selective blockade of HCN channels by 60 μM ZD 7288 reduces inward currents, but perhaps not completely or some subunits are still activated, since at −125 and −115 mV steps there are gradually increasing currents with a latency of ~1 s ^[30]^. The ZD 7288 effects on both sag currents and potentials are heterogeneous and also in terms of required concentrations ^[19]^. The magnitudes of tails are a better indicative of the potency of ZD or any other drugs on HCN channels (Figure 5). Although tails are truncated, as expected, 55 mM ethanol did not obliterate them ^[30]^.

It is considered that alcohol contributes to anxiety and CVT-10216 – a selective inhibitor of aldehyde dehydrogenase-2 (ALDH-2) may have an opposite effect by reducing the ethanol intake by P70 rats ^[31]^. This study proves again that any change in brain function is complex as people drink moderately in order to socialize, but CVT-10216 prevents alcohol consumption in control animals and promotes the social interaction in anxiety prone Fawn-Hooded rats.

The NMDA receptor mediated glutamate neurotransmission is oppositely modulated be ethanol and nicotine. Ethanol inhibits the amplitude of evoked excitatory postsynaptic currents (EPSCs) in the CA1 area of the hippocampus in P12–20 rats ^[32]^. On average the effects were significant only at 75 mM ethanol (despite the decreased amplitudes at 25 and 50 mM) that is similar in respect to miniature EPSCs. As mentioned before, also in this preparation the paired stimulation of Schaeffer collaterals at 50 ms interval results in heterogeneous PPR – paired pulse ratio (in 2 cells facilitation – PPF while in 4 the depression – PPD). After the ethanol only PPF are observed. When the interval was increased to 100 ms the PPR did not change in one cell while in 3 the PPD were enabled and ethanol increased the ratio in all. Nevertheless, in one neuron with the largest PPD ethanol did not lead to PPF and the ratio barely reached the value of 1. Therefore, it does not reflect the notion that the ‘second of two synaptic stimuli delivered in rapid (typically, 20–200 ms) succession will be larger than the first due to transient presynaptic Ca^2+^ loading’ ^[32]^. Ethanol decreased also the inter-event intervals, but the presence of data points for −100 ms (even with the values of 0) under control condition is not clear.

The P – alcohol preferring rats differ not only in behavior, but also in active properties of VTA DA neurons ^[33]^. Despite the similar spiking rates at ~4 Hz, an increase in bursting (*n* = 88 *vs*. 57) and percentage of SWB (50 *vs*. 34) were indicated compared to ‘a general population of Wistar rats’ ^[33]^. High, low and medium preference for alcohol by ~P114 rats has been distinguished with respective intakes of more than 65, <25 and 25–65% of total fluid ^[34]^. In a medium range, rats receiving a daily 1 mg/kg s.c. nicotine ditartrate salt increased their ethanol intake and preference by ~160 and 180%. The intake decreased to ~125% after 45 days of withdrawal. The value of preference is higher, since rats were getting addicted to ethanol independent of nicotine. Rats increased their preference to ~130% after only 7 days of enabling a choice between alcohol and water. The withdrawal effects were observed also in respect to preference. Despite the intensive behavioral research one question remains unanswered ‘why does the ligand nicotine have dedicated receptors, but addictive ethanol does not?’ ^[1]^.

### Amphetamine

Amphetamine similar to apomorphine and L-dopa was found to prevent epileptic discharges evoked by electrical stimulation of caudate nucleus ^[35]^. Indirect effects of amphetamine also on synapses were shown by electrocorticogram and waves occurring specifically at ~100 Hz ^[36]^. Amphetamine (i.v.) at 0.06 mg/kg has dual effects on male rat VTA DA neurons excitability as it decreases in majority, while increases in a minority ^[37]^. Under similar conditions, 0.005 mg/kg apomorphine only decreased the spike frequencies. When rats were primed with 1 mg/kg s.c. amphetamine twice during 24 h for 6 days, similar tendencies in the effects of i.v. amphetamine and apomorphine persisted. However, at 5 mg/kg s.c. amphetamine the dual effects were proportional. In control rats 2 mg/kg i.v. amphetamine decreases the neuronal activity by ~90% and when primed with 1 and 5 mg/kg s.c. amphetamine the respective values were ~60 and 50%. Thus, the dual effects was demonstrated by a ‘paradoxical increase in VTA activity produced by d-AMPH in 4 control rats’ ^[37]^ that was absent in SNc. The rate of spiking was ~3 Hz under control conditions. Note that, since the frequencies of VTA DA neurons are heterogeneous, four different modes were distinguished in P21 WT mice ^[38]^. The rate of spikes in WT and α7^−/−^ mice were comparable at ~4 Hz, while in β2^−/−^ it decreased to ~2 Hz.

The antipsychotic drug cyamemazine at 5 μM started to accumulate into puncta after 10 min of exposure in rat SNc as revealed by two-photon microscopy *in vitro* ^[39]^. Thereafter it increased gradually and reached the peak at the 50 min time point. The fluorescence intensity of cyamemazine insignificantly increased to ~4% during triggered trains of spikes at 10 Hz. However, although the ΔF value is ~0% (but how it can have a SEM), it is not clear why the cyamemazine should respond in control group? Interestingly, 50 mM K^+^ decreased the accumulation by ~7% and it is unexpected as this procedure is to some extent equivalent to somatically evoked APs.

There is also a possible interaction with other drugs of abuse as the study involving trained male rats for i.v. methamphetamine self-administration points to ^[40]^. The results revealed a dose dependent decrease in intake by CB_1_ antagonist AM 251 (1–5 mg/kg). A ~60% decrease was revealed and these effects were achieved when 15 min prior to nose poke operandum 2 mg/kg AM 251 was i.p. injected. Under identical conditions and concentration the agonist anandamide increased the intake by ~115% but no significance was estimated. Even ~125% increase with 5 mg/kg R-(+)-methanandamide was considered not significant.

Although it is obvious that any drug including the amphetamine may have several targets, but their effects perhaps is linked to a single molecule ^[41]^.

### Cannabis

#### CNS

This drug is more extensively studied, since it has receptors unlike the ethanol, and moreover there is an endogenous ligand in contrast to nicotine. A simple DSE and DSI experiments enable readout of the retrograde transmission into the presynaptic neurons. However, these phenomena are not robust, at least not the DSE in amygdala ^[11]^.

In humans cannabinoids exert a wide range of action spanning between tachycardia and impairment of memory. Their effects are long lasting at least on human cognition that persists after withdrawal. The Δ^9^-tetrahydrocannabinol (THC) is considered as the key ingredient of marijuana. The cannabis abuse by smoking marijuana can be determined by routine urine immunoassay screening test. Interestingly, there were cases doubting the outcomes of this test, since the chocolate also contains anandamide-like constituents. However, there is no cross reaction between these N-oleoyl- and N-linoleoylethanolamide of chocolates and N-arachidonylethanolamide in urine ^[42]^.

The i.v. injection of 15 mg anandamide in castrated ~P35 male calves under non-stress conditions immediately increases the serum cortisol from ~5 to 15 ng/ml ^[43]^. Within 20 min it reaches a plateau with values of ~35 ng/ml and after remaining at this level for 30 min it gradually decreases to the baseline during ~1 h. Note that the pre-drug values differ significantly because of ‘some unknown difference in the physiological state of the calves prior to injection’ ^[43]^. WIN 55212-2 had very similar effects on cortisol levels and all phases and values were comparable.

Using an antibody and peptide blocking approach it was found that the receptor GPR55 is activated by the cannabinoid ligand CP55940 ^[44]^. The *E*_max_ of CP55940 for CB_1_, CB_2_and GPR55 receptors were similar at ~100%, while that of anandamide were respectively 66, 58 and 73%. The contents of the ligands arachidonylethanolamide and 2- arachidonylglycerol (2-AG) could be precisely determined in clinical tissue samples by gas chromatography-mass spectrometry-selected ion monitoring ^[45]^. However, since 2-arachidonylglycerol is spontaneously isomerised to biologically inactive 1-AG, the studies/diagnoses dealing with 2-AG contents in tissue samples should be taken with precaution ^[46]^.

CB_1_ receptors are differentially located in the CNS of rats and are altered in AE – acquired epilepsy – model ^[47, 48]^. In the majority of nuclei there were either a significant or non-significant increase (e.g. ~2 fold in septum), but not a decrease ^[48]^. In interneurons of the CA1 area of the hippocampus the muscimol-evoked GABA_A_ currents were either significantly decreased or not affected by 30 nM N^6^-cyclopentyladenosine (CPA) that correlated with the presence and the absence of CB_1_R, respectively [49]. This study reveals the interaction of CB_1_R with the adenosine receptor A_1_as CPA is an agonist.

In lower vertebrates the dynamic function of CB_1_ receptors is evident in both the CNS and periphery. The role of CB_1_R in the development of *Xenopus laevis* is also implicated. Already during organogenesis the CB_1_Rs are expressed in these animals ^[50]^. The mammalian ortholog of CB_1_ and CB_2_ receptors have been also discovered in goldfish whose telencephalic and the inferior lobes of the posterior hypothalamic brain areas exhibit CB_1_-like immunoreactivity ^[51]^. The eCBs were detected in entire brain areas of goldfish. The food deprivation for 24 h led to the selective elevation of anandamide, but not 2-AG in the telencephalon. The CB_1_Rs are also present in the avian brain and its receptor recognition site is similar to those of mammals ^[52]^. The embryonic chick brain expresses the CB_2_-like receptors, which were absent in adult animals. The anandamide and palmitoylethanolamine are also present in lipid extracts of bivalve mollusks ^[53]^. Their contents increase when mussels are extracted 24 h post-mortem, but remain unaffected by boiling the tissue prior to extraction.

#### Anterograde neurotransmission

An addiction to cannabinoids is not the only concern as there are more complications that accompany it including the impairment of memory. DSE – a form of short term memory is modulated by eCBs. By relatively brief depolarization may be enabled whether or not retrograde signaling is present between the post– and presynaptic neurons. The release of endocannabinoids in postsynaptic cells may occur with several paradigms, perhaps not necessarily only with depolarization *in vitro* ^[54]^. The depolarizing paradigms also differ and some represent the close physiological conditions than the others ^[11, 55]^.

In striatal medium spiny neuron (MSN) the DSI is induced by 5 s steps to 0 mV from a HP of −80 mV ^[56]^. As mentioned before retrograde signaling is hard to manifest *in vitro* ^[11]^ and therefore in order ‘to induce discernible DSE’ ^[56]^ the identical 80 mV steps were applied in the presence of 50 μM DHPG, the type 1 metabotropic glutamate receptor (mGluR1) agonist. Even under these conditions, only ~30% DSE was observed compared to the DSI of ~40%. The diacylglycerol (DAG) lipase blocker tetrahydrolipstatin decreased them respectively to ~2 and 5%.

In CA1 area of the hippocampus of male rats both AM 251 and SR141716 (a selective CB_1_ cannabinoid receptor antagonist) at identical concentrations of 1 μM increase the slope of – field excitatory postsynaptic potentials – fEPSPs in similar manner ^[57]^. Although the effects appear impressive on the graph, the values are only ~107% of baseline at peak. During the concurrent application of AM 251 with either 10 μM bicuculline or 1 μM CGP55845 the similar increases were observed. The slope was decreased to ~70% of baseline by 20 μM meloxicam and these effects were reversed by subsequent concurrent AM 251 to ~94%. The AM 251 was applied during 20 min, but perhaps even longer time will not enable a complete reversal as effects have reached a plateau. This already hinted at a partial CB_1_ independent effect, which was confirmed by adequate experiments, i.e. the simultaneous application of AM 251 and meloxicam. Unlike in amygdala ^[11]^ in this preparation a clear cut PPF is observed when 50 ms intervals are chosen ^[57]^. Consistently the amplitude of fEPSPs are significantly deceased by meloxicam at P1 resulting in ~18% increase in PPF that almost completely was reversed by AM 251. Thus, the cyclooxygenase-2 (COX-2) but not COX-1 was considered a responsible molecule for the modulation of retrograde signaling in the hippocampus. The authors, under identical conditions, with the same animals also elucidated the mechanism of LTP ^[58]^. Again the recording site was the stratum while stimulation was targeted to Schaffer collaterals. After recording fEPSPs during 10 min a train of 100 pulses at 100 Hz were triggered that led to an immediate potentiation of ~200%. The latter has gradually decreased and resulted in LTP of ~125% of baseline after 1 hour. During the intense induction with two trains the respective values were ~210 and 150% and under both conditions 1 μM AM 251 was ineffective. Interestingly, the AM 251 facilitated the LTP when it was induced by a single train of 50 pulses at 100 Hz (109 *vs*. 132%). There could be also some additive effects of priming as the same slices were stimulated beforehand with trains of 10 and 20 pulses at 100 Hz and AM 251 had an influence during those episodes. An increase was observed also when the LTP was induced by theta burst stimulation paradigms.

Among studies the waveform of fEPSPs often differs exhibiting a prominent rebound depolarization in rats of similar ages, at least the maximal ones as judged by their comparable BM of 180 *vs*. 170 g ^[58, 59]^. Nevertheless, both phases are reversibly decreased by 10 μM anandamide even after its 1 hour long application ^[59]^. The complete effects of wash-out also took about the same time, i.e. recovery was slow and gradual. These author have recorded comparable PPF and made complex analyses as ‘at the end of the anandamide application, the stimulation intensity was increased to counteract a direct depressant effect of anandamide’ ^[59]^ on the amplitude of fEPSP at P1. Interestingly, the rebound phase is greater according to the amplitude of fEPSP at P2. They have observed PPF at all tested intervals of up to 200 ms and anandamide facilitated it further. It shifts the input-output response curve of the postsynaptic spike and fEPSP to the right. AEA and WIN 55,212-2 suppress the epileptic activities in the CA1 and CA3 areas of the hippocampus, which could be experimentally induced by either omission of the Mg^2+^ or hyperkalemia (8 mM K^+^). SR141716 prevents the abnormality, which is consistent with the notion that endocannabinoids are involved in retrograde tuning of neuronal excitability and/or excessive activity under pathophysiological conditions.

The AEA content in the brain could be altered by introducing the same substance exogenously ^[60]^. However, priming with phenylmethylsulfonyl fluoride (PMSF), an inhibitor of amidohydrolase, is required. Thereby, a 16-fold increase in the brain concentration of AEA was achieved. The AM-374 blocks the metabolism of anandamide into arachidonic acid and ethanolamine by the enzyme anandamide amidase ^[61]^. The AEA content increases with the development in the striatum ^[62]^. However, the level of 2-AG during the postnatal period remained unchanged. The content of AEA is also increased already within 30 min of transient middle cerebral artery occlusion (MCAo) in the injured ischemic side and after 60 min in both hemispheres ^[63]^. It has been shown that the WIN55,212-2 decreases the PPD in the CA1 area of the hippocampus ^[64]^. The activation of CB_1_Rs decreases the GABA_A_ release in the hippocampus. The 2-AG has similar effects on PPD at a concentrations ranging from 1 to 30 μM. The identical concentrations of anandamide, however, increased PPD, that was not reversible by the cannabinoid receptor antagonist AM-281.

The ‘emotionality’ in P70 females rats was probed with behavioural tests ^[65]^. Only the open field test resulted in sufficient alteration of time spent in the centre by 10 μg estradiol – from 22 to 34 s that was prevented by 1 mg/kg AM 251. Changes in immobility and struggling times were concluded to be non significant that proves a need for the departure from statistics and back to basic discoveries with the ‘n-of-one’ experiment ^[66]^. Respective values after estradiol were 65 *vs*. 108 and 101 *vs*. 150 s ^[65]^. At least the SEM values of immobility time were similar at 20 *vs*. 26 s. Therefore, the ‘potential importance of endocannabinoid signaling in emotional behavior in females’ ^[65]^ specifically is not plausible.

#### Retrograde neurotransmissions – DSE and DSI

DSE is infrequently studied, but it is observed even in autaptic neurons ^[67]^. Unlike VTA and many other nuclei, the hippocampus is recognized at P0–2 in mice and neurons of CA1 and CA3 are distinguished from other regions in this nucleus. The highest tested concentration of CB_2_ agonist JWH015 (2 μM) inhibited evoked EPSCs by ~46% and effects reached the plateau at this level. Interestingly, 200 nM SR141716 restored the amplitude to 100% during about 2 min application. However, the average data reflects only ~86%, therefore perhaps ‘it was fully reversed’ ^[67]^ only in one neuron. AM630 similarly affected the eEPSCs and effects were not dose dependent when comparing 2 *vs*. 10 μM. When PP were applied, as mentioned before ^[26]^, it did not result in clear cut PPF as expected from the 60 ms interval, but only in two neurons ^[67]^. In remaining 11 neurons there were PPD of varying degrees. Identical concentration of JWH015 specifically decreased the PPR in those two neurons, but in others increased or was ineffective yielding a neutral PPR of ~1 *vs*. 0.8. Traces are not adequately presented and reader can not appreciate the magnitude of P1 and P2 EPSCs under control and JWH015 conditions. Note that the autaptic EPSC has a slower rising phase compared to a conventional one. These autaptic neurons even after 50 ms depolarization to 0 mV from a holding potential of −70 (not +70) mV exhibit DSE with the maximum of ~50% during 10 s. Although neurons were tested after additional 8 DIV, they are still immature and can not be considered representative of pyramidal neurons or drug targets. It should be emphasized that many regions of the brain in rodents (Figure 2) are straight forward to spot compared to humans.

DSE has been observed in proopiomelanocortin (POMC) neurons of the arcuate nucleus (ARC) of hypothalamus of P50–75 male Topeka guinea pigs ^[68]^. POMC neurons are considered ‘anorexigenic’ in this study, but ‘cells responsible for appetite’ suffice. They state also the BM of animals, but it should be emphasized that the properties of any neurons strictly correlate with the age, but not weight, at least under control conditions. The frequencies of mEPSCs are high with values more than 1 Hz whether guinea pigs were treated with sesame oil or 400 μg TP – testosterone propionate. This might not be related to orchidectomy. WIN at 30 nM affected the cumulatively frequency of mEPSCs in TP group only, but the mean rates were decreased in both. WIN only at 30 nM differentially affected mEPSCs rates, but between 100 nM and 3 μM effects were similar in both groups with the maximal inhibition of ~50%. TP alone increased the frequencies of mIPSCs from ~4 to 14 Hz. If analyzed one could learn that also amplitudes are increased in the TP group. DSE of vehicle groups is small, but that of TP robust and is blocked by 1 μM AM251 (perhaps in a different cell, since frequencies before the depolarization also differ). DSE was probed with 3 s 60 mV step from HP of −75 mV, and since the depicted duration of traces are only ~20 s after depolarization (apparently to −15 mV) no sign of recovery could be appreciated. Note that the DSE or DSI of spontaneous events are also transient and often very short. Although the study has dealt with males, neurons, and testosterone, but at least this part of results is hard to connect to ‘sex difference-based therapeutic adjuncts for use in ameliorating the symptoms associated with HIV/AIDS and cancer’ ^[68]^, but everything in nowadays science is possible.

In SNr – *pars reticulata* nucleus of substantia nigra (mainly GABAergic neurons) DSI (10 s, from −70 to 0 mV) is observed, but suppression is relatively long lasting compared to CA1 and complete recovery is achieved at ~3 min ^[69]^. DSI of SNc DA neuron was on average smaller. In both nuclei AM251 at 1 μM concentration prevents DSI and decreases the amplitude variation of evoked inward currents. There was also a tonic release of eCBs as AM251 enhanced the amplitude of eIPSCs in both nuclei. It was concluded that inputs into both types of neurons contain CB_1_ and D_1_ receptors.

Although, changes in retrograde signaling occur robustly during only several seconds, if it takes place recurrently, it may have a distinct impact on addictive behaviors. Note that the retrograde neurotransmission is shown for CB_1_, since CB_2_ cannabinoid receptors are prevailingly established in the periphery, but not in CNS. Zhang and colleagues ^[70]^ delivered a strong message that not only CB_1_, but also CB_2_ are present and may modulate brain area involved in addiction. Some of data are presented in perhaps unfortunate manner and diminishing their importance. Since representative experiments are ‘illustrating that CB_2_R staining was detected in VTA DA neurons in WT and CB1^−/−^ mice, but barely detectable in CB2^−/−^ mice’, yet average values are ‘illustrating a significant reduction in the density’ ^[70]^. Density of ~8 *vs.*19 is n ot after all ‘barely’.

It is not clear which panels were merged, and why the intensity of DAPI is changed. Also figure 1S is not ‘related to Fig. 2’ ^[70]^, but identical and merged panels from. CB_2_ mRNA is localized to soma (figure 2A), while it does not clearly appear in merged panel (figure 2B). CB_2_R densities in CB2^−/−^ are almost identical despite different numbers of neurons (~400 *vs.* 200). This is hard to achieve, unless, antibodies are from a single source. Only ~50 % of CB_2_R are downregulated in CB2^−/−^, yet APs are unaffected by 1 μM JWH133 (figure 4C). Will 10 μM have any effects? It is rather a disadvantage that all five agonists have similar potency.

Dopamine is responsible for anterograde, while endocannabinoids, for retrograde neurotransmission ^[11]^. Since there is no tonic release of cannabinoids, agonists are often used. But JWH133 effects are not convincing ^[70]^, since APs in VTA DA neurons do not always have stable rate and variability started right before the application. Spikes often occur with bursting pattern ^[70]^, rate oscillates ^[71, 72]^, and as a pioneer study shows ^[73]^, frequency could be lower. This view is supported by the fact that after longest period of inactivity ^[70]^, the neuron fires with closely graded double spikes (figure 4B), which is an intrinsic property ^[20]^. JWH133 effects on RMP are significant ^[70]^, but it is well documented that also these values for VTA DA neurons can vary greatly. In the presence of JWH133 only one AP was triggered (figure 4D), which is not sufficient for the frequency calculation. Also recordings of spontaneous spikes are short and do not reflect ‘after stable recording for several minutes (baseline), JWH133 or other drugs were bath-applied to the recorded neuron for 1–2 min and then washed out’ ^[70]^. Recording and analyses in figure 4D are important, but many values do not match averages (*Table S2*). AHP calculation is wrong and not compensated for changes in RMP. Why should JWH133 hyperpolarize RMP, unless it is affecting ion channels? Note there are no changes in RMP by JWH133 during spontaneous activity. There is bursting at time points when spikes are blocked by quinpirole. AP phenotypes are different in figure 4D and *F*, hinting that the heterogeneity applies to all major properties of VTA DA neurons.

#### Peripheral nervous system

Although the expression of CB_1_Rs is mainly shown in the CNS, nevertheless these receptors are detected also at the peripheral level. Thus, the CB_1_Rs are not only present in the myenteric plexus-longitudinal muscle (MPLM) of the guinea-pig ileum, but also modulate the adenosine release ^[74]^. The electrically evoked release can reach ~150 or 250 nM as detected by an adenosine sensor in two control groups. The former was decreased to ~25 nM by 10 nM CP55,940. Application of 100 nM SR141716 slightly increased the release, as it did concurrently also with CP55,940, but both these changes were not significant. Interestingly, the activation of CB_1_Rs in the myenteric neurons decreases the gastrointestinal (GI) motility in ileal longitudinal muscle-myenteric plexus (LMMP) preparations ^[75]^. The effects of WIN 55,212-2 and AEA depend on the age of animals, however, the expression of CB_1_R in these neurons is age independent.

In male rats the delivery of 20 μg capsazepine (CAPZ) via intrathecal catheter decreases the paw withdrawal (PWD) latency from ~9 to 7.5 s in the non-inflamed side ^[76]^. There were no latency changes in the inflamed side and this procedure decreases the PWD to a peak and plateau values of ~3 and 5 s, respectively. AEA at 100 μg increased its plateau value to ~9 s and thus decreased the hyperalgesia for the entire tested duration of ~1 h. Authors caution that 100 μg AEA ‘also caused temporary vocalization and excitation, suggesting a pain-inducing potential of AEA’ ^[76]^. The following is not clear in regard to these two separate experiments: ‘1^st^ and 2^nd^ arrows show the injection of CAPZ/vehicle and AEA/vehicle, respectively’. When injected concurrently capsazepine prevented the effects of AEA by ~50% at abovementioned concentrations. Thus, in the spinal cord the AEA is antinociceptive that is realized through the activation of TRPV1 receptors. Effects of AEA and WIN 55,212-2 have been studied in regard to the experimentally induced central pain syndrome in rats ^[77]^.

#### Receptor channels

The anandamide may bypass the CB_1_R signalling pathway and act independently, at least *in vitro*. Thus, the anandamide blocks the current activated by kainic acid in *Xenopus* oocytes expressing GluR3 – type 3 AMPA glutamate receptor ^[78]^. In this expression system SR141716A did not reverse the effects of anandamide. Interestingly, the SR141716A in a mouse model of anxiety causes anxiolysis and inhibits the anxiogenic effects of anandamide *in vitro*. These inconsistent findings under *in vivo* and *in vitro* conditions are not surprising since the oocytes do not posses the intra– and intercellular signalling cascades as in native cells. For example, GluR3 may require other auxiliary subunits in order to function similar to those of native neurons, thus the CB_1_R function is much more complex.

The triggered release of GABA and glutamate in hippocampal synaptosomes are decreased by 1 μM WIN and these effects are prevented by CPA at 100 nM concentration ^[79]^. It is common that either the activation of one receptor type may influence another one or they interact ^[80]^. Moreover, a single substance may target multiple receptor and channel types as it could be exemplified by zinc ^[81]^.

WIN55,212-2 at 1 μM almost abolishes the 30 μM serotonin evoked inward currents at −100 mV in HEK 293 cells expressing h5-HT_3A_ receptors ^[82]^. However, its enantiomer WIN55,212-3 was ineffective under identical conditions and concentration. Even 0.3 μM WIN55,212-2 was potent and an reversible blocker of theses receptors and did not affect the serotonin dose-response curve though the magnitude of currents at tested concentration ranged between 1 and 100 μM. Note that WIN55,212-2 was effective only when applied before serotonin, and even simultaneous application of both did not prevent the inward current.

The *IC*_50_ values of arachidonylethanolamide and the plant THC for the inhibition of prostaglandin E_1_-stimulated adenylate cyclase activity in Chinese hamster ovary-K1 (CHO-K1) cells transfected with the cannabinoid receptors are ~10 and 100 nM, respectively ^[83]^.

In rat cortical microglial cultures TNFα release is achieved by bacterial endotoxin lypopolysaccharide (LPS) in a concentration dependent manner ^[84]^. Between each logarithmic concentration a ~2 fold increase in TNF was observed resulting in ~ 0.5, 1 and 2 ng at 0.1, 1 and 10 μg/ml LPS. The effects of native (AEA and 2AG) and synthetic (WIN 55,212-2 and HU210) cannabinoids are dose-dependent and occur in a range of 1–10 μM with different affinities. Among these drugs only HU210 and WIN obliterated the release at 5 and 10 μM, respectively. These glia cells express CB_1_ and CB_2_receptors.

Despite the established access to neurons of mammals and skepticism over non-native cells, oocytes are used widely as a platform, especially in studies concerning mutations. When possible it is more adequate to study targets in native neurons as certain effects are observed only in one expression system, but notin another ^[85, 86]^.

#### Ion channels

Ca^2+^ channels, especially those active around the resting membrane potential, modulate the excitability of neurons. The mibefradil sensitive Ca^2+^ channels therefore are classified as low voltage-activated (LVA or T-type) and are encoded by Ca_v_3.1, Ca_v_3.2 and Ca_v_3.3. The Ca2+ currents through the latter α-subunits are abolished by anandamide ^[87]^. AEA directly blocks these channels, which modulate the pacemaker activity of neurons, especially during epilepsy ^[88]^. This blockade occurs at the cytoplasmatic side of cell membrane, since the inhibition of anandamide membrane transporter AM-404 antagonizes the AEA effects. The AEA also directly blocks the P-type Ca^2+^ channels and increases the inactivation rate in Purkinje neurons with the *IC*_50_ of 1 μM ^[89]^. The AEA metabolism may not underlie the effects seen on P-type Ca^2+^ currents, since Met-AEA had similar effects. The inhibition by these endocannabinoids was not reversed by antagonists of both CB_1_ and TRPV1 – transient receptor potential vanilloid type 1. WIN 55,212-2 modulates spontaneous firing of neurons in cerebellar slices despite the presence of CB_1_ receptor antagonists substantiating the existence of other receptors or unrelated targets.

The expression of CB_1_Rs has been revealed in cat cerebral arterial smooth muscle cells (VSMC), which interact with L-type Ca^2+^ channels ^[90]^. The anandamide inhibits the binding of 1,4-dihydropyridine, 1,5-benzothiazepine and phenylalkylamine to L-type Ca^2+^ channels ^[91]^. Thus, endocannabinoids block the Ca^2+^ influx through Ca^2+^ channels and thereby regulate the cerebral vascular tone.

### Cocaine

This compound, when delivered (15 mg/kg i.p.) multiple times, inhibits the HCN channels in VTA DA neurons of adult rats recorded *in vitro* and effects appear to be selective ^[92]^. Upon the return to a HP of −60 mV from −130 mV hyperpolarizing step a robust *I*_h_ tail currents are activated that do not decay completely during ~500 ms (Figure 5). In rats sensitized to cocaine the peak tail currents are also blocked, but the magnitude of final decay remained unaffected and is similar to that of saline treated animals. HCN currents waveform is comparable to LS neurons including that of tails ^[19]^ despite the differences in HP of −60 *vs*. −70 mV. The major distinction between the LS and VTA DA neurons is the activation threshold of −100 *vs*. −80 mV, respectively ^[92]^. The tails and RAP are interrelated, since 10 μM ZD 7288 obliterates them ^[19]^. Depending on the amplitude of sag there could be also only the RTP instead of RAPs. In two groups the values of tail currents were used also for determination of reversal potentials (*E*_rev_) that were indistinguishable and occur at about −39 mV ^[92]^. However these might not be adequate, since A: at −60 mV the *I*_h_ tail was robust, but this MP was not considered for *E*_rev_ protocol, and B: values were estimated in a complex way yielding −11 pA at −50 mV step, but the current magnitude should be ~0 pA as its peak value (coincides with measured point) is similar to that of a holding current at −60 mV. An appropriate tail protocol could be similar to those as shown in DRG neurons at a more negative MP ^[93]^.

The *IV* relationship of evoked AMPA currents in VTA DA neurons of P18-22 mice is almost ideally linear (even considering that values are normalized) when measured between −70 and +40 mV ^[94]^. The latter is not strictly linear in i.p. 15 mg/kg cocaine injected mice based on distributions of data points. This difference was considered unique and the authors ‘suggested that in cocaine-treated mice, some AMPARs were lacking the GluR2 subunit’ ^[94]^. The observed ‘rectification’ in cocaine group contradicts some findings of the established literature as at least in MS there are both rectifying and non-rectifying AMPA currents ^[95]^. Specifically, authors contradict their prior results as AMPA currents ‘showed strong rectification at positive potentials and were sensitive to Joro spider toxin (JST), a selective blocker of GluR2-lacking AMPARs. After mGluR-LTD, AMPAR EPSCs had linear current-voltage relations and became insensitive to JST’ ^[96]^. Of note, the amplitude dynamics of data points under control conditions in this case are more realistic.

The JST at 500 nM selectively decreased (in MP dependent manner) the EPSC in cocaine group ^[94]^. Effects started to develop immediately and gradually increased during ~20 min, and it reached a plateau at +40 mV, but not at −50 mV with a respective decrease of ~70 and 45% of baseline. The value with a negative polarity of ‘−7.0 ± 7.3% in naive mice’ ^[94]^ perhaps means an increase. In control mice 20 μM DHPG was ineffective, but led to LTD (therefore mGluR-LTD) in the cocaine group. Effects of DHPG similar to that of 66 Hz stimuli were abrupt. Interestingly, the persisting suppression during ~25 min was considered as LTD that is not commonly accepted for an LTP ^[97]^. Between the two studies there is inconsistency also with regard to DHPG as it originally induces LTD but not later ^[94, 96]^. This discrepancy could not derive from differences in species used, i.e. mouse *vs*. rat since all other conditions were either identical or similar.

Rodents not only develop a preference for alcohol, but they can distinguish cocaine over the vehicle. The rats at ~P80 ‘over the weekends’ ^[98]^learned to press a lever in order to receive an infusion of cocaine (i.p. and each time 0.5 mg/kg). At an identical dose of 10 mg/kg gabapentin insignificantly increased cocaine self-administration, but tiagabine decreased it significantly as did 150 mg/kg vigabatrin. Interestingly, the number of infusions significantly decreased only in case of vigabatrin. Why do the numbers of lever presses and infusion not correlate well?

Even a simple behavior in rats could be complex as upon the initial introduction to a lever they press multiple times at higher frequencies. They perhaps learn later that pressing just one time is enough to receive desired fluids. In P90–120 male rats it was observed that after a 1 month of abstinence they similarly attempt to receive a cocaine infusion (0.33 mg) as before, i.e. ~20 times per 2 h ^[99]^. There was correlation in behavior of these rats during the distinct phases of addiction and the excitability of neurons in the NAc. Cocaine self infusion changes the plasticity in NAc, but does not alter the neurotransmitter parameters ^[100]^. The mean amplitude of spontaneous EPSCs were ~12 pA in all three groups (control, addicted and resistant rats), that makes one wonder how big the magnitude of miniature events would be? Also from cumulative probabilities it appears that the first detectable events have amplitudes of ~10 pA. The inter-event intervals can not be judged, since their cumulative probabilities are not adequately presented. In all groups also PPF was similar with values of ~150%. The latter is robust when compared to identical 50 ms intervals between the two pair of pulses in amygdala ^[11]^. The spontaneous and evoked events are very homogenous ^[100]^. Note that the path to addiction (in those vulnerable) was very short and took 17 days at most after the self exposure to cocaine.

In ‘predominately Caucasian (75.6%), male (77.8%) and never married (53%)’ cocaine addicts the effects of tiagabine at two doses (12 *vs*. 24 mg/day) were tested ^[101]^. Some of these patients have consumed cocaine during more than 3 days each week according to their self-report, and after a high dose of tiagabine it was reduced by 42%. However, it is not clear why the placebo group should reduce cocaine use by 21%? If participants are Caucasians it should be considered as a limitation of this study? Interestingly, reduction by a low dose of tiagabine was ~55%. Tiagabine is used also in a lower concentration of 4 and 8 mg as these doses are effective in treatment of epileptic patients ^[102]^. These two doses (in reference to 0 mg) did not robustly change effects of oral use – discrimination of 150 mg cocaine by participant compared to a placebo – at least as one can judge by graph data. All ‘participants selected between a chance to receive the drug from that day or a gift card to a local grocery store. The dollar value of this gift card increased from US$ 0.25 to US$ 64 across the nine choices’ ^[102]^. However, there are discrepancies in numbers alone in one figure: ‘data for the 4mg tiagabine group represent the mean of four participants and data for the 8mg tiagabine group represent the mean of six participants. Data are the average of the five-point distribution determinations conducted during each experimental session and represent the mean of six participants’ ^[102]^. Why do some of these points possess SEM while others lack it?

Both a drug seeking and fear response are perhaps triggered by PFC albeit via different nuclei ^[103]^. This study at least proves that not only the brain but each nuclei are complex in terms of anatomy, function, and role. Anatomically it is really hard to distinguish between Cg1, DP, IL, PL – cingulate, dorsal peduncular, infralimbic, prelimbic nuclei of mPFC, at least in brain slices used for electrophysiology (Kodirov, unpublished). Therefore, these ‘four major subdivisions of rodent medial prefrontal cortex are depicted along the Paxinos and Watson anatomical boundaries’ ^[103]^. During *in vivo* experiments foci could be retrospectively traced by sectioning the brain with microtome. Based on responses to electrical stimulation it was summarized that Cg1 and PL enhance cocaine-seeking and fear, while DP and IL decrease them.

Similar to cannabinoids ^[9]^ also cocaine influences the *vas deferens* and at 10 μM facilitates the 20 μM NA induced contraction (via α_1_-adrenergic receptors) by ~2 fold [104].

### Heroin

There is a certain curiosity as to the effects of recreational drugs and the state of mind in humans. Some dedicated scientists in history extended their curiosity also to dangerous procedures and substances as ‘recently four of us used ourselves as subjects in an experiment with heroin. It was very unpleasant’ ^[105]^.

Heroin effects on brain can be revealed even after 5 min of infusion (200 μg/kg i.v.) that alters CA2, DG, MS, SNc, and SNr, but not amygdala, CA1, CA3, CA4 and VTA activities in rats ^[106]^. In a different group of rats cocaine (1 mg/kg i.v.) altered the majority of these regions, but not the amygdala, CA1, and DG. All changes are small and have different polarities. In heroin addicts also changes in multiple regions including the VTA DA neurons are predicted, specifically after the withdrawal ^[5]^. Changes in brain activities by heroin withdrawal become more severe when addicts also concurrently abuse alcohol ^[10]^. As stressed previously humans may consume several drugs of abuse and interestingly, even a single substance may have overlapping effects involving multiple targets as shown for MOR – mu opioid receptor and the NE (NA) – norepinephrine (noradrenalin) circuit in heroin self-administering male rats ^[107]^. Heroin increased the interaction of MOR to WLS (MOR-interacting protein Wntless) by ~140% of baseline in the *locus coeruleus* (LC). Note that WLS was expressed not only in LC, but also in the hippocampus, mPFC, NAc, striatum, and VTA.

Specific changes in SGZ –subgranular zone of human DG has been documented postmortem after ~2 days of heroin intoxication ^[108]^. In addicts neurons positive to Musashi-1 has decreased (despite one outlier) in numbers to ~10 *vs*. 17% in controls. Effects (not 0.4 but 40% should read the scale) did not correlate with the age in addicts, but significant correlation was observed in controls. The latter difference is perhaps derived by longer maximal age spans in controls up to ~40 *vs*. 30 years. This study is insightful in terms of view point as ‘without a doubt, it is important to compare animal data to humans’ ^[108]^ and how the total parameters of heroin addicts matched those of controls in regard to age, average age, age spans, brain weight, and gender. Addict females were younger than males with mean values of ~20 *vs*. 27 years. Their respective mean brain weights were slightly lower. The most perfect match was in mean brain weights of males in addict and control groups with almost identical value of ~1469 g as their SEM were not too apart. Some addicts were concurrently abusing alcohol and morphine.

### Morphine

Direct exposure of VTA DA neurons to morphine led to the reinstatement of i.v. heroin self-administration in rats and these effects were prevented by naltrexone ^[109]^. Although this review highlights the obvious no discrimination by brain areas, drugs, interactions, and targets ^[1]^, it appears that effects in this case were selective as ‘morphine applied to several other brain areas rich in opiate receptors does not reinstate the behavior’ ^[109]^.

The subcutaneous exposure to 150 mg morphine for up to 6 days results in addictive behavior in rats ^[110]^. Morphine increased the frequency of spikes in VTA DA neurons from ~4.3 to 5.9 Hz *in vivo*. Clonidine (0.1 mg/kg i.p.) does not affect spike properties, but changes the polarity of naltrexone (0.1 mg/kg i.v.) effects from inhibitory to excitatory. The naltrexone when applied alone decreases the rate of spikes in morphine rat model, but 10 min subsequent to clonidine it increases the frequency by ~150% of baseline. Even in this case an initial effect is inhibitory although transient. Interestingly, the excitatory effect is also accompanied by an increase in amplitudes of spikes (by ~0.4 mV) that is reflected almost in a symmetrical manner to both depolarizing and hyperpolarizing phases of spikes. The latter occurs gradually and after ~1 min reaches the peak and thereafter declines at the same pace. The statement that ‘firing rate remained above the baseline up to 60 min after the naltrexone injection’ ^[110]^ is not strictly correct as effects were significant only within the first ~20 min. As clonidine is the agonist of α_2_ adrenergic receptors the involvement of NE was concluded ^[26, 110]^.

One of the insightful studies demonstrated the effects of morphine (1 mg/ml, total volume of 60 nl) by a direct delivery into the VTA ^[71]^. Morphine increased frequencies of single spikes and bursts of VTA DA neurons and effects correlated with the basal rates of individual cells with lowest in faster spiking neurons (~8 Hz). Effects are progressive after the infusion during ~5 min. Its magnitude was higher when comparing with the i.v. delivery that is more physiological. The effect of i.v. morphine was prevented when either 100 μM naltrexone or 100 μM AP5 and 50 μM CNQX were infused into the VTA beforehand. There were a weak correlation between frequencies of both spikes and bursts in DA neurons recorded from different regions of VTA with respective values of ~4, 5, 6 and ~0.6, 1, 1 Hz. The spike waveforms were similar in these cells. Interestingly, infusion of 1 mM picrotoxin (60 nl) also increased the rates of both spikes and bursts in VTA DA neurons. Morphine under identical conditions decreases the spike rate of GABAergic neurons from ~13 to 5 Hz. It explains that effects of morphine on DA neurons are not direct, but occur via GABAergic cells located in the tail of VTA. Although the frequencies of GABAergic neurons are higher and reach ~25 Hz, the rates in lower range overlap with those of DA ones.

### Nicotine

Consumption of cigarettes triggers complex cascades in human CNS and other organs. Exposure to nicotine not only targets nACh receptors throughout the brain but may lead to a release of dopamine including those from the VTA. The dopamine release by nicotine in the striatum of rats is facilitated by ghrelin – a growth hormone releasing peptide ^[111]^. Ghrelin may also independently trigger the DA release. It has been suggested that ghrelin may modulate food intake (the reward source) by affecting nAChRs ^[112]^. Plasma levels of ghrelin perhaps is dynamic depending on food intake but it was not apart when diets of two eggs and one portion of oatmeal were compared ^[113]^.

Nicotine increases DARPP-32 (dopamine- and cAMP-regulated phosphoprotein of Mr 32 kDa) Thr34 phosphorylation only during 1^st^ 30 s and thereafter it declines ^[114]^. Although, it was stated that ‘treatment with nomifensine alone did not affect the level of phospho-Thr34 DARPP-32’ ^[114]^, but bands representing 10 and 100 μM nicotine experiments are faint compared to 0.1 and 1 μM at 0 time points. Perhaps therefore, effects of 1 μM nicotine were significant at 3 and 5 min while those of higher concentration not.

Effects in P12–45 mice are strictly not dual ^[19]^, but could be considered as heterogeneous, since in 1 out of 9 neurons of laterodorsal tegmental – LDT area no effects and in another 10 μM nicotine decreased the number of APs despite the increase in mean values ^[115]^. Influences were targeted to overall excitability only, since the amplitude and waveforms of APs are similar including those of AHP.

Nicotine may directly target ion channels as shown for HCN that is responsible for membrane potential properties including the sag ^[19]^. The phenotype of sag in neurons differs depending on region were they reside. In SNc DA neurons of male P14–28 rats the time course of the sag is fast and the steady-state is reached within ~1 sec ^[39]^. The 1^st^ rebound AP lack AHP in contrast to subsequent two spikes. The AHP is either masked by the depolarizing phase of the RTP or it is a double spiking as observed in LS neurons ^[20]^. Spontaneous spikes in SNc DA neurons occur in a pacemaker manner and possess AHP ^[39]^. The GABAergic cells of SNc trigger ‘a pure sodium spike, while dopamine neurons have a major contribution of calcium current to their spikes. Of course, there is variability between individual neurons’ (Dr. E. S. Levitan, personal communication). The double spiking occurs also during the spontaneous activity when primed with nicotine ^[20]^. The second spike in a doublet originates from an ADP after its enhancement by nicotine.

Published ‘n-of-one’ study revealed that nicotine patch almost obliterated epileptic attacks that had irregular frequencies with the maximum of 10 occurrences per day ^[66]^. During double blinded placebo observations this maximum reached only 5 and it perhaps occurred less since the patient in the prior ~ 8 months was under the exposure of a nicotine patch. Possibly similar studies in combination with imaging approach will yield more clear cut outcomes depending on the type of both techniques and epilepsies ^[116]^. The seizure in turn may occur in parallel with other pathologies and involve the same targets ^[117, 118]^.

## Conclusions

Scientists made great progress by using rodents, and it is a good thing that we are not involving dogs in addiction science, although it is hard to digest studies on human being in general, and those requiring the insertion of microelectrodes into the brain in particular ^[8, 119]^.

There are no differences in basic behavior among mammals if and when they are prone to addiction as ‘humans and laboratory animals given access to opiate and stimulant drugs frequently become compulsive users of these drugs, and often, in spite of prolonged periods of abstinence, persist in drug-seeking behavior and relapse to drug-taking’ ^[109]^.

Addiction establishes itself gradually via multiple phases and has components, of which maybe the withdrawal is most powerful. Addictions are also perhaps similar to other syndromes is accompanied by unrelated pathologies of CNS ^[120]^. I think in one of these studies this notion was best summarized: ‘withdrawal of many drugs is associated with a rebound increase in paradoxical sleep, both in terms of its duration and in terms of its intensity, the latter manifested by increased profusion of eye movements and greater intensity of the dreams’ ^[105]^.

Therefore, it will be proved that none of the known ^[121]^ and yet unknown compounds will have the one and only one target on the cell membrane or its compartments as revealed earlier, e.g. for 9AA –aminoacridine ^[122]^.

## Abbreviations

ADP: after-depolarizing potential
AEA: arachidonylethanolamide
AHP: after-hyperpolarizing potential
AMPA: α-amino-3-hydroxy-5-methyl-4- isoxazolepropionic acid
AP: action potential
BLA: basolateral amygdala
CA1: *cornu ammonis* (subdivision 1 out of 4 within the hippocampus)
CB_1_: cannabinoid receptor type 1 (CB_2_ – type 2)
D_1_R: dopamine receptor type 1 (there are more including the DRD2)
DSE: depolarization-induced suppression of excitation
DSI: depolarization-induced suppression of inhibition
eCB: endogenous cannabinoids
EPSC/P: excitatory postsynaptic current or potential
GABA: γ-aminobutyric acid
GluR2: ionotropic glutamate receptor type 2 (mGluR1– metabotropic)
HCN: hyperpolarization-activated cyclic nucleotide gated non-selective cation channel
IPSC/P: inhibitory postsynaptic current or potential
LA: lateral amygdala
LS: lateral septum
LTD: long term depression (synaptic)
LTP: long-term potentiation
MP: membrane potential
mPFC: medial prefrontal cortex
MS: medial septum
nAChR: nicotinic acetylcholine receptor
NMDA: N-methyl-D-aspartate receptor
PFC: prefrontal cortex
PPD: paired pulse depression
PPF: paired pulse facilitation
PPR: paired pulse ratio
RAP: rebound action potential
RMP: resting membrane potential
RTP: rebound tail potential
SR141716: N-(piperidin-1-yl)-5-(4-chlorophenyl)-1-(2,4-dichlorophenyl)-4-methyl-1H- pyrazole-3-carboxamide
SWB: spikes within a burst (the numbers of AP)
TNF: tumor necrosis factor
VTA: ventral tegmental area (*area tegmentalis ventralis*)
WIN 55,212-2: R(+)-[2,3-dihydro-5-methyl-3-[(morpholinyl) methyl] pyrrolo [1,2,3-de]-1,4-benzoxazinyl]-(1-naphthalenyl) methanone mesylate.
